# Patienteneinwilligungen für das TraumaRegister DGU® aufgrund der EU-Datenschutz-Grundverordnung (EU-DSGVO) – Eine Herausforderung für die Kliniken: Status quo und Lösungsstrategien

**DOI:** 10.1007/s00113-021-01060-0

**Published:** 2021-07-28

**Authors:** T. Herbst, D. Popp, C. Thiedemann, V. Alt, A. Ernstberger

**Affiliations:** 1 Klinik und Poliklinik für Unfallchirurgie, Universitätsklinikum Regensburg, Franz-Josef-Strauß-Allee 11, 93053 Regensburg, Deutschland; 2 Klinikum Osnabrück, Am Finkenhügel 1, 49076 Osnabrück, Deutschland

**Keywords:** Registerforschung, Einwilligungsquote, Schwerverletztenversorgung, Überregionales Traumazentrum, Datenschutz, Research database, Response rate, Multiple trauma care, Level I trauma center (supraregional), Data privacy

## Abstract

Gemäß der Datenschutz-Grundverordnung (EU-DSGVO, Mai 2018) werden anonymisierte Datensätze mit ausreichend hoher Datendichte als nachverfolgbar eingestuft und benötigen eine Einwilligungserklärung, wenn diese zu Forschungs- oder Qualitätskontrollzwecken zentral ausgewertet werden. Qualitätssicherung und weitere Steigerung der Versorgungsqualität sind im Rahmen der flächendeckenden Schwerstverletztenerhebung im Sinne der Versorgungsforschung allerdings nur mit einer annähernden Vollerhebung möglich. Die über 600 deutschen Kliniken, die am TraumaRegister DGU® teilnehmen, versuchen, von diesem speziellen Patientengut die Einverständniserklärungen zu erhalten. In der Studienklinik wurden über einen 12-Monats-Zeitraum hinweg die Rate an Einwilligungen und die Gründe für eine Ablehnung bzw. Nichteinholung evaluiert.

Bei Anwendung eines ressourcenintensiven Workflows speziell für die Patientenaufklärung und Einholung der TR-Einwilligungen wurden eine Zustimmungsquote der Patienten von 64,5 % und damit gleichzeitig eine Fehlquote von 35,5 % erfasst. Es konnten 98 von 276 potenziellen TraumaRegister-DGU®-Datensätzen nicht eingegeben werden und standen dementsprechend weder für die Qualitätskontrolle noch für die Polytraumaforschung zur Verfügung.

Um die Qualitätskontrolle und die weitere Verbesserung der Versorgungsqualität zu gewährleisten, ist eine annähernde Gesamterfassung des Patientenguts notwendig. Diese lässt sich durch die Notwendigkeit der Einwilligungserklärung jedoch nicht erreichen, wie unsere Studie zeigt. Somit plädieren wir dafür, dass die Möglichkeit geschaffen wird, den TraumaRegister-Datensatz ohne Einwilligung zu erheben, da dieser letztlich einen Regeldatensatz darstellt, vergleichbar mit dem §21-KHEntgG-Datensatz, jedoch im Gegensatz zu diesem pseudonymisiert.

## Abstract (en)

According to the General Data Protection Regulation (GDPR 05/2018), anonymized data sets with a sufficiently high data density are classified as traceable and require a declaration of consent if they are evaluated centrally for research or quality control purposes. Quality assurance and further increases in the quality of care are, however, only possible with a nearly complete survey of seriously injured persons in the sense of health services research. The more than 600 German clinics that take part in the TraumaRegistry DGU® try to obtain the declarations of consent from this special patient population. The study clinic evaluated the rate of consent and the reasons for rejection or failure to obtain consent over a 12-month period. While using a resource-intensive workflow especially for patient education and obtaining the consent, a patient consent rate of 64.5% and an error rate of 35.5% were recorded. Of the 276 potential TraumaRegistry DGU® data records 98 could not be entered and were therefore neither available for quality control nor for multiple trauma research. In order to guarantee the quality control and the further improvement of the quality of care, an approximate total recording of the patient population is necessary; however, this cannot be achieved by requiring a declaration of consent. We therefore advocate creating the possibility of collecting the TraumaRegistry data set without consent, as this ultimately represents a standard data set, comparable to the Hospital Remuneration Act (§21-KHEntgG) data set but pseudonymised.

## Herausforderung „Patienteneinwilligung“ für das TraumaRegister DGU®

Seit Inkrafttreten der EU-Datenschutz-Grundverordnung (EU-DSGVO) im Mai 2018 mit verschärften Verantwortlichkeiten und Haftungsbedingungen werden Datensätze, die bisher als anonymisiert oder pseudonymisiert galten, alleine aufgrund der Datendichte als nachverfolgbar angesehen. Auch die Daten des TraumaRegister DGU® (TR) sind hiervon betroffen. Über 600 am TR teilnehmende Kliniken versuchen, Einwilligungen Schwerverletzter zur Weitergabe von Regeldatensätzen an einen zentralen Forschungsserver einzuholen. In der vorliegenden retrospektiven „Single-center“-Studie wurde die Rücklaufquote über 12 Monate hinweg erfasst und evaluiert.

## Material und Methode

Die Studienklinik ist als Universitätsklinikum der einzige Maximalversorger in einem ländlich geprägten Gebiet mit ca. 2 Mio. Einwohnern und etwa der Fläche von Hessen. Von den ca. 700 Traumapatienten, welche in dieser pro Jahr im Schocktraum behandelt werden (GoR‑A und GoR-B), entsprechen ca. 300 Patienten dem Basiskollektiv des TraumaRegister DGU®, ca. 150 Patienten weisen einen Injury Severity Score (ISS) ≥ 16 auf. Aufgrund dieser hohen Fallzahl wurden 12 Studierende angestellt, um in 24/7-Rufbereitschaft die Dokumentationsarbeit im Schockraum zu leisten und nach Ende der Verweildauer den Fall – bei vorliegender Einverständniserklärung – ins TraumaRegister zu übertragen. Anschließend erfolgen die Fallkontrolle (Vollständigkeit/Richtigkeit) und Freigabe (endgültiger Fallabschluss) durch die TR-beauftragten Ärzte der Klinik für Unfallchirurgie.

Die TR-Einwilligungserklärung wird in der Studienklinik im Verlauf des stationären Aufenthalts via persönlicher Individualaufklärung des Patienten/gesetzlich Bevollmächtigten eingeholt. Von den an der Studienklinik diskutierten und letztlich verworfenen Möglichkeiten der Einholung der Einwilligungserklärung ist v. a. die Kopplung an den Patientenvertrag erwähnenswert. Dies war aus internen logistischen und auch rechtlichen Gründen nicht umsetzbar. Insbesondere wird der Patientenvertrag bei schwerstverletzten Patienten ggf. primär nicht bzw. nicht vom Patienten/gesetzlich Bevollmächtigten unterschrieben.

Die Einholung der Individualaufklärung wird nun an der Studienklinik durch einen aufwendigen, aber strukturierten und möglichst effektiven interdisziplinären Workflow abgebildet, mit dessen Hilfe alle Traumapatienten, die den Einschlusskriterien des TraumaRegister DGU® entsprechen, aufgeklärt und um ihre Einwilligung zur Datenübertragung ins TR gebeten werden sollen: Die Traumapatienten erreichen die Klinik über die interdisziplinäre Notaufnahme (Schockraum) und werden organisatorisch meist unfallchirurgisch geführt. Die stationäre Aufnahme nach operativer Versorgung erfolgt vorwiegend auf 2 der insgesamt 6 Intensivstationen (interdisziplinär operativ und neurochirurgisch) bzw. auf 4 definierten Normalstationen (Unfallchirurgie und Neurochirurgie). Die Einholung des Einverständnisses erfolgt auf den genannten Stationen durch die dortigen Case-Manager bzw. Pflegekräfte.

**Figure 1:**
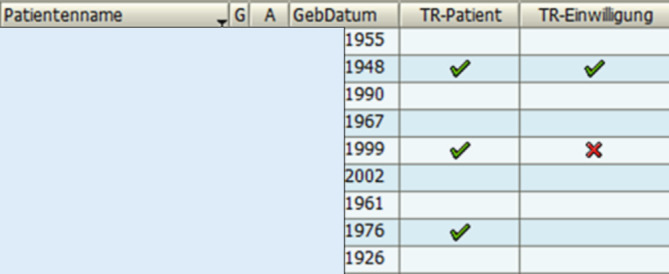

Im Klinikinformationssystem (SAP) wurden von der IT-Abteilung 2 Spalten generiert: „TraumaRegister-Patient“ und „TraumaRegister-Einwilligung“ (Abb. 1). Patienten, welche die TR-Einschlusskriterien erfüllen, werden im Schockraum bzw. spätestens nach der Frühbesprechung am Folgetag identifiziert und gleichzeitig in der SAP-Spalte „TR-Patient“ durch einen Arzt oder die Studienkoordinatorin mit grünem Häkchen markiert. Sobald ein Patient entsprechend markiert ist, wissen die Pflegekräfte und Case-Manager der betreffenden Intensiv- und Normalstationen, dass dieser im Verlauf des stationären Aufenthalts über das TR aufzuklären und ihm die Einwilligungserklärung vorzulegen ist. Die Einwilligung wird entweder vom Patienten selbst unterzeichnet, sofern dieser körperlich und geistig dazu in der Lage ist, oder von seinem gesetzlichen Betreuer.

Liegen Patienten außerhalb der oben genannten chirurgischen Stationen, werden sie durch die diensthabenden Stationsärzte der Unfallchirurgie aufgesucht und über das TR aufgeklärt. Sobald das Einwilligungsformular unterschrieben ist, wird in der SAP-Spalte „TR-Einwilligung“ ein weiteres grünes Häkchen gesetzt. Wird die Teilnahme am TR aktiv abgelehnt bzw. verweigert, so ist dies durch ein rotes X gekennzeichnet. Fällt bei einer ambulanten Nachuntersuchung auf, dass ein Patient bislang weder eingewilligt noch abgelehnt hat, so wird dieser nachträglich aufgeklärt und um Einwilligung gebeten. Die unterschriebenen Einwilligungsbögen werden zentral in der Unfallchirurgie gesammelt.

In die vorliegende Studie eingeschlossen wurden Patienten, welche zwischen dem 01.08.2019 und dem 31.07.2020 verunfallten und die im KIS als TR-Patient markiert worden sind. Zuverlegungen direkt auf die Intensivstation ohne Schockraumversorgung, Verbrennungen, Verbrühungen, Strangulationen, Commotio-Patienten mit anschließender Überwachung auf der Intensivstation sowie reine Beckenregisterpatienten, die ebenso in den TR-Einwilligung-Workflow einbezogen sind, blieben bei der vorliegenden Studie unberücksichtigt.

Signifikante Unterschiede wurden für *p* < 0,05 angenommen. Für die Berechnung wurden Chi-Quadrat-Test und t‑Test herangezogen.

## Darstellung der Ergebnisse

276 Patienten konnten in die Studie eingeschlossen werden. Davon sind 22 verstorben. Insgesamt konnte von 178 Patienten (64,5 %) eine Einwilligungserklärung eingeholt werden (Tab. 1). Betrachtet man ausschließlich die 254 überlebenden Patienten, beträgt der Prozentsatz 70,1 %.

**Table 1:** 

Gesamtstichprobe mit Rücklaufquoten *(auf eine Nachkommastelle gerundet)*	*n*	%
**TR-Fälle (12 Monate) *n*** **=** **276**	**276**	**100**
Einwilligung vorhanden	178	*64,5*
Einwilligung nicht vorhanden	98	*35,5*
**Davon ISS ≥** **16**	**140**	**50,7**
Einwilligung vorhanden	82	*58,6*
Einwilligung nicht vorhanden	58	*41,4*
**Davon Basiskollektiv**	**136**	**49,3**
Einwilligung vorhanden	96	*70,6*
Einwilligung nicht vorhanden	40	*29,4*
**Einwilligung nicht vorhanden *n*** **=** **98**	**98**	**100**
Verstorben im SR oder im Verlauf des stationären Aufenthalts	22	*22,5*
Intensivstation eines anderen Fachbereichs (Ø UCH, NCH)	22	*22,5*
Normalstation eines anderen Fachbereichs (Ø UCH, NCH)	19	*19,4*
Zeitnahe Ab- bzw. Weiterverlegung	9	*9,2*
Teilnahme am TR aktiv verweigert/abgelehnt	8	*8,2*
Sprachbarriere (weder Deutsch noch Englisch, kein Dolmetscher vor Ort)	6	*6,1*
Nicht einwilligungsfähig (bewusstlos, intubiert, desorientiert u. Ä.)	5	*5,1*
Frühe Entlassung gegen ärztlichen Rat	3	*3,1*
Grund retrospektiv nicht ermittelbar	4	*4,1*

Mit Blick auf die Gesamtfehlquote von 35,5 % über den Studienzeitraum von 12 Monaten hinweg wurden die Gründe dafür, dass 98 potenzielle TR-Patienten nicht ins Register Eingang finden konnten, evaluiert und aufgearbeitet:

### Verstorbene Patienten

Von 98 Patienten ohne vorliegende Einwilligung verstarben 22 Patienten im Schockraum bzw. im unmittelbaren Verlauf. Gemäß Artikel 1 Abs. 1 der DSGVO i. V. m. Erwägungsgrund Nr. 27 der DSGVO gilt die Datenschutz-Grundverordnung nicht für personenbezogene Daten Verstorbener [3]. Diese Patienten könnten somit nach dem Tod auch ohne vorliegende Einwilligungserklärung und ohne Einwilligung der Angehörigen in das TR eingetragen werden. Jedoch besteht noch keine endgültige Rechtssicherheit darüber, ob damit ggf. gegen die ärztliche Schweigepflicht verstoßen werden würde [9]. In Hamburg und Brandenburg gilt der Datenschutz auch über den Tod hinaus [6]. Aus diesem Grund wurden im Rahmen der vorliegenden Studie die verstorbenen Patientinnen und Patienten in der Gruppe der Patienten ohne Einwilligung belassen.

### Fachfremde Unterbringung und sonstige Gründe

22 Patienten (*22,5* *%*) wurden auf einer fachfremden Intensivstation (z. B. Pädiatrie, Innere/Kardiologie) und 19 Patienten (*19,4* *%*) auf einer fachfremden Normalstation untergebracht. Ursächlich hierfür waren, neben speziellen Traumadiagnosen (z. B. MKG-Chirurgie) und dem Alter des Patienten (Pädiatrie), auch kardiologische oder sonstige Vorerkrankungen oder auch Kapazitätsauslastungen der unfallchirurgischen Stationen.

*9,2* *%* der fehlenden Einwilligungen betrafen Patienten, die rasch ab- bzw. weiterverlegt wurden. 8 Patienten (*8,2* *%* der *n* = 98 fehlenden Einwilligungen bzw. *2,9* *%* aller *n* = 276 potenziellen TR-Patienten) lehnten die Datenübertragung ins TraumaRegister aktiv ab. *6,1* *%* der Patienten waren aufgrund einer Sprachbarriere (weder deutsch- noch englischsprachig) nicht adäquat aufzuklären, *5,1* *%* waren aufgrund von Bewusstlosigkeit, Intubation, Desorientierung o. Ä. nicht einwilligungsfähig mit fehlendem Kontakt zum gesetzlichen Betreuer, *3,1* *%* verließen die Klinik frühzeitig entgegen ärztlichem Rat, und bei *4,1* *%* der Patienten waren die Gründe für die fehlende Einwilligung retrospektiv nicht mehr ermittelbar.

### Verletzungsschwere

Es wurde untersucht, ob ein Zusammenhang zwischen fehlenden Einwilligungserklärungen und der Verletzungsschwere bestand: 140 von insgesamt 276 Patienten waren schwerstverletzt mit einem ISS ≥ 16. Von dieser Gruppe konnte in *58,6* *%* der Fälle die Einwilligung eingeholt werden; in *41,4* *%* der Fälle gelang dies nicht. Die übrigen 136 Patienten des Basiskollektivs (ISS < 16) zeigten eine Einwilligungsquote von *70,6* *%,* in *29,4* *%* konnte keine Einwilligung eingeholt werden. Die Einwilligungsquote ist im Basiskollektiv signifikant höher als bei den schwerstverletzten Patienten mit ISS ≥ 16 (*p* = 0,037).

### Durchschnittliche Verweildauer

Betrachtet man die Verweildauer, zeigte sich auch hier ein Einfluss auf die Rücklaufquote der TR-Einwilligungen (Tab. 2). Alle 178 Patienten, von denen das Einverständnis rechtzeitig während des stationären Aufenthalts eingeholt werden konnte, wiesen mit durchschnittlich *15,6* Tagen signifikant (*p* < 0,001) längere Verweildauern auf als die 98 Patienten, von denen die Einwilligung im Verlauf des stationären Aufenthalts nicht eingeholt werden konnte (*8,4* Tage).

**Table 2:** 

Ø Verweildauer (*n* = 276)		Ø Verweildauer 0 bis 5 Tage (*n* = 71)	
Einwilligung vorhanden (*n* = 178)	Einwilligung nicht vorhanden (*n* = 98)	Einwilligung vorhanden (*n* = 26)	Einwilligung nicht vorhanden (*n* = 45)
*15,6*	*8,4*	*3,3*	*2,1*
*P* < 0,001		*p* = 0,002	

71 der insgesamt 276 Patienten waren nur 5 Tage und weniger stationär an der Klinik. 26-mal konnte hier eine Einwilligung erwirkt werden (Ø Verweildauer *3,3* Tage); bei 45 Fällen (Ø Verweildauer *2,1* Tage) ist dies nicht gelungen (*p* = 0,002).

## Diskussion

Echte, explizite Verweigerungen (*n* = 8, *8,2* *%* der Patienten ohne EV-Erklärung) waren in unserer Studienstichprobe selten. Das Gros der Patienten und auch der Angehörigen/Betreuer stand einer Datenübertragung in das TraumaRegister DGU® sehr aufgeschlossen gegenüber. Die sehr geringe Anzahl von aktiven Verweigerungen könnte darauf zurückzuführen sein, dass das TraumaRegister auf Regeldatensätzen basiert und keine darüber hinaus gehenden Untersuchungen des Patienten erforderlich sind. Auch eine qualitativ gute Aufklärungsarbeit (fachlich fundiert, patientengerecht und einfühlsam) kann dazu beitragen, die Einwilligungsquote der Patientinnen und Patienten zu erhöhen [1, 4]. Die Bereitschaft, durch reine Datenübertragung aktiv an der Verbesserung künftiger Schwerverletztenversorgung mitzuwirken, ist bei sachlicher, adäquater Information und Aufklärung grundsätzlich groß und wird selten infrage gestellt. An fehlender Compliance der Patienten sollte ein Forschungsprojekt wie das TraumaRegister letztlich nicht scheitern.

Problematisch ist vielmehr, den Patienten überhaupt während des stationären Aufenthalts erreichen zu können, um entsprechende Aufklärungsarbeit zu betreiben und die Einwilligung einzuholen. Als besonders schwierig erwies sich die Aufklärung bei Patienten, die auf fachfremden Stationen an der Studienklinik untergebracht waren und dementsprechend nicht durch die bereits entsprechend geschulten Case-Manager aufgeklärt werden konnten. Auf fachfremden Stationen oblag diese Aufgabe den unfallchirurgischen Stationsärzten. Die Einwilligung konnte in 19 Fällen (≙ *19,4* *%* aller fehlenden Einwilligungen) auf fachfremden Normalstationen nicht eingeholt werden; auf fachfremden Intensivstationen betrug die Fehlquote sogar *22,5* *%*. Die Studie zeigt, dass die TR-Aufklärung im klinischen Arbeitsalltag der Ärzte nicht immer umsetzbar war. Die tägliche Visite bietet kaum ein Zeitfenster für eine zusätzliche, adäquate Aufklärung. Ein hohes Patientenaufkommen oder Zeiten mit dünn gestrickter Personaldecke führen zwangsläufig zu den dargestellten Fehlquoten, da der Patientenversorgung unbedingt Vorrang vor organisatorischen Aufgaben gewährt werden muss. Ferner spielt die Verweildauer eine Rolle, denn sobald ein Patient nur 5 Tage oder weniger stationär im Klinikum verweilt, erfolgten Aufklärung und Einwilligung kaum mehr rechtzeitig während des Klinikaufenthalts.

Im Zuge der Coronapandemie wurden einige Strukturen und standardisierte Abläufe an der Studienklinik verändert, sodass die Workflow-Routine während dieser Krisenzeit mancherorts nicht mehr greifen konnte. Limitierte Besucherregelungen haben im 1. HJ 2020 dazu geführt, dass Erziehungsberechtigte, Angehörige oder Betreuer nur selten am Krankenbett angetroffen wurden. Personelle Engpässe durch Abordnungen von Ärzten und Pflegepersonal auf die COVID-19-Stationen, urlaubsbedingte Vertretungssituationen und Sonn- und Feiertage führten ebenso dazu, dass der etablierte Workflow nicht mehr routiniert und reibungslos griff.

Weiterhin liegt die Vermutung nahe, dass schwerstverletzte Patienten (Kriterium ISS ≥ 16) aufgrund ihres körperlichen oder psychisch belasteten Zustands schlechter in der Lage sind, ihre Einwilligung zu äußern als weniger schwer verletzte Patienten. DGOU-Präsident Grützner befürchtete in diesem Zusammenhang während des DKOU 2019, das Bild des TraumaRegisters verzerre sich, wenn nur noch die Daten weniger kritisch verletzter Patienten einfließen [10]. Die Zahlen unserer Studie zeigen, dass *58,6* *%* der schwerstverletzten Patienten mit ISS ≥ 16 ihre Einwilligung während des stationären Aufenthalts erteilten. Der Anteil vorliegender Einwilligungen lag mit *70,6* *%* in der Gruppe ISS < 16 signifikant höher. Wenn 58 von 140 Patienten mit ISS ≥ 16 nicht in das Register eingegeben werden können, darunter auch die verstorbenen Patienten, ist ein verzerrtes Abbild der Klinikrealität nicht von der Hand zu weisen.

Das primäre Ziel der Schwerstverletztenforschung ist die Minimierung der Mortalität. Dementsprechend ist der primäre Endpunkt der Untersuchung das Überleben/der Tod eines Patienten. Würde man alle Verstorbenen aus dem Register herausnehmen, verlöre die Polytraumaforschung letztlich die Möglichkeit, ihrer Bestimmung nachzukommen [11].

Die gesetzliche Verpflichtung der Leistungserbringer zur Qualitätssicherung ist im § 135a SGB V regelt. Das TraumaRegister DGU® ist eine etablierte, einrichtungsübergreifende und flächendeckend in allen TraumaNetzwerken DGU® gelebte Qualitätssicherungsinitiative und für die teilnehmenden Kliniken ein wichtiges Instrument zur gesetzlich geforderten internen und externen Qualitätssicherung. Die Teilnahme am TR ist somit in erster Linie keine wissenschaftliche Studie, für die eine Einwilligung zu Recht und ohne Ausnahme gefordert werden muss, sondern v. a. Qualitätssicherung. Eine solche kann nur erfolgreich sein, wenn die Datengrundlage vollständig vorhanden ist, sodass die Realität möglichst exakt abgebildet wird. Die Vollständigkeit der Daten ist im TraumaRegister aktuell jedoch vom guten Willen des Patienten abhängig, wodurch dem Register ein beträchtlicher Anteil an Informationen wegbricht. Die Registerdaten entsprechen damit nicht mehr der Versorgungsrealität und können – vielmehr dürften – nicht mehr länger als Indikator für die klinische Versorgungsqualität herangezogen werden. Relevante Qualitätssicherung aus dem TR heraus ist somit nicht mehr möglich. Insbesondere Artikel 9 der DSGVO [3] bezieht sich auf die Verarbeitung besonderer Kategorien personenbezogener Daten. Ausnahmeregelungen wären möglich, allerdings müssten die europäischen Mitgliedsstaaten diese in ihre Gesetzgebung übernehmen. In Deutschland ist eine solche Ausnahme für das TR derzeit nicht gesetzlich verankert. Externe Qualitätssicherung – basierend auf Routinedaten – muss wieder legal ermöglicht werden; gerne mit engmaschiger Überprüfung oder Zertifizierung des externen Datenhalters. Wenn Forschung und Fortschritt im Bereich der Schwerverletztenversorgung noch länger ausgebremst werden, wird die Polytraumaforschung in Zukunft nicht mehr auf dem gewohnt hohen Level möglich sein.

## Resümee

Initial rechnete man an der Studienklinik nicht annähernd mit einer Einwilligungsquote von *64,5* *%*. Allerdings konnte diese Quote nur mit dem hohen Ressourcenaufwand eines Universitätsklinikums und höchst engagierter, interdisziplinärer Zusammenarbeit unterschiedlichster Mitarbeitergruppen erarbeitet werden. Sie ist dennoch in Anbetracht der damit einhergehenden Fehlquote von *35,5* *%* höchst unbefriedigend: Über ein Drittel der Patienten des betrachteten ÜTZ fehlen im TraumaRegister DGU®, weil die Patienteneinwilligungen nicht vorlagen; darunter auch die Daten der Verstorbenen, die der Forschung nun keine Erkenntnisse mehr zur Verbesserung der Schockraumversorgung werden liefern können [7, 9].

Die positive Entwicklung der Schwerverletztenversorgung der vergangenen Jahre basiert einerseits auf Forschungsergebnissen, welche maßgeblich aus dem TraumaRegister DGU® generiert wurden [5, 14]. International anerkannte Leitlinien der höchsten Qualitätsstufe zur Schwerverletztenversorgung wurden hieraus erarbeitet und laufend angepasst.

Andererseits beruht die hohe Versorgungsqualität auf dem (intrinsischen) Qualitätsanspruch der Traumazentren. Teilnehmende Kliniken erhalten regelmäßig Feedback zu ihren Leistungszahlen im anonymen Benchmark-Vergleich, um Verbesserungspotenzial zu erkennen und geeignete Maßnahmen zur Verbesserung der Patientenversorgung ergreifen zu können [12]. Diese Aspekte führten nach und nach in Traumazentren aller Versorgungsstufen und damit in den Traumanetzwerken zu einer flächendeckenden Verbesserung der Versorgungsqualität und besserem Outcome für schwer verletzte Patienten [2, 13].

Möchte man weiterhin ein reelles Abbild der Traumaversorgung in Deutschland für Forschung und v. a. für die Qualitätssicherung in den einzelnen Kliniken erhalten, ist dies nur mit einer annähernden Vollerhebung möglich. Unsere Studie zeigt, dass trotz höchstem Einsatz an Ressourcen diese nicht zu erreichen ist, wenn die persönliche Einwilligungserklärung des Patienten (letztlich zur Weitergabe eines vorhandenen Regeldatensatzes) notwendig ist [6].

Eine Möglichkeit wäre, eine gesetzliche Grundlage zur Datenerhebung zu schaffen. Als Beispiele können das Bundeskrebsregisterdatengesetz (BKRG) oder das Implantateregistergesetz (IReG) dienen. Der Bayerische Landtag hat am 28.05.2020 [8] einem Antrag zweier Parteien zugestimmt und damit befürwortet, dass sich auch die bayerische Staatsregierung für die Schaffung einer Rechtsgrundlage für das TR auf Bundesebene einsetzen solle. Es bleibt zu hoffen, dass der Bund dieser Forderung entspricht, damit die wissenschaftliche Qualität des TraumaRegister DGU® [15] auf Dauer wieder uneingeschränkt und unverfälscht existieren kann und das TR seine seit 1993 bestehende, wichtige Funktion als wertvolles Qualitätssicherungsinstrument [5] für die Schwerverletztenversorgung langfristig behalten kann. Zu fordern ist hierbei jedoch, dass die Datenhoheit in den Reihen der Fachgesellschaft bleibt. Hierdurch können die freie Forschung und das hohe Potenzial durch das Erreichen einer kritischen Masse an Forschungsprojekten einerseits, andererseits die objektive Qualitätssicherung gewährleistet werden. Die Datenauswertung des TraumaRegister DGU® wird ständig modifiziert, angepasst und lebt von neuen Ideen. Ein starres Korsett würde diesem Register ebenso schaden wie die Patienteneinwilligungserklärung.

### Die vorliegende Studie zeigt verschiedene Limitationen

- Es handelt sich um eine Single-center-Studie, welche in einem Universitätsklinikum durchgeführt wurde. Der personelle und monetäre Aufwand, welcher für die Einholung der Einwilligungserklärungen betrieben wurde, kann nicht für alle Kliniken in Deutschland als repräsentativ gelten. Es ist davon auszugehen, dass eine flächendeckende Auswertung der fehlenden Einwilligungen einen höheren Prozentsatz als die beschriebenen *35,5* *%* erbringen würde.
- Das Studiendesign war retrospektiv. Dieser prinzipielle Nachteil verhinderte jedoch unserer Meinung nach einen „Erfüllungsbias“. Es konnte nicht zu besonders geführten Aufklärungsgesprächen wegen der Studie kommen.
- Die Zuteilung der Verstorbenen in die Gruppe der nicht ins TR eingegebenen Patienten erscheint sehr restriktiv, entspricht jedoch der aktuellen Rechtsgrundlage, welche sich nicht eindeutig darstellt.

## Fazit für die Praxis

- Trotz eines ressourcenintensiven, interdisziplinären Workflows konnte im Verlauf von 12 Monaten nur von knapp 65 % der potenziellen TR-Patienten eine Einwilligung eingeholt werden.
- Über ein Drittel der möglichen TR-Patienten, darunter aufgrund der unklaren rechtlichen Situation auch alle Verstorbenen, konnten wegen fehlender Einwilligungen nicht in das TraumaRegister DGU® eingetragen werden. Dies beeinträchtigt die Datenqualität v. a. hinsichtlich der Vollzähligkeit stark.
- Weniger als 3 % aller TraumaRegister-Patienten lehnten über den Studienzeitraum von 12 Monaten hinweg die Datenübertragung ins TR aktiv ab.
- Die Einwilligungsquote ist in der Gruppe ISS < 16 signifikant höher als bei Schwerverletzten mit ISS ≥ 16.
- Je länger die Verweildauer, desto höher die Chance, den Patienten über das TR aufzuklären und die Einwilligung zu erhalten.
- Die Schaffung einer gesetzlichen Grundlage, pseudonymisierte TR-Daten ohne vorherige Einwilligung der Patienten zu sammeln und in die Registerdatenbank einzustellen, wäre eine Möglichkeit, um die Datenqualität langfristig zu sichern.

## Abkürzungen

DGOU: Deutsche Gesellschaft für Orthopädie und Unfallchirurgie
DGU: Deutsche Gesellschaft für Unfallchirurgie
DKOU: Deutscher Kongress für Orthopädie und Unfallchirurgie
EU-DSGVO: EU-Datenschutz-Grundverordnung
KIS: Krankenhausinformationssystem
TR: TraumaRegister DGU®
ÜTZ: Überregionales Traumazentrum
